# Transcultural adaptation and validation of a Korean version of the Oxford Ankle Foot Questionnaire for children

**DOI:** 10.1186/s12955-020-01378-0

**Published:** 2020-05-01

**Authors:** Seong Hee Cho, Chin Youb Chung, Moon Seok Park, Kyoung Min Lee, Ki Hyuk Sung

**Affiliations:** 1 Department of Orthopaedic Surgery, Gyeongsang National University Hospital, 79 Gangnam-ro, Jinju, Gyeongsangnam-do 52727 Republic of Korea; 2 Department of Orthopaedic Surgery, Seoul National University Bundang Hospital, 82 Gumi-ro 173 Beon-gil, Seongnam, 13620 Gyeonggi Republic of Korea

**Keywords:** Oxford ankle foot questionnaire for children, Korean, Translation, Transcultural adaptation, validation

## Abstract

**Background:**

The purpose of this study was to translate and transculturally adapt the original English version of the Oxford Ankle Foot Questionnaire (OAFQ) into a Korean version, and to evaluate its psychometric properties.

**Methods:**

A Korean OAFQ for children was developed according to established guidelines. To test validity, 169 consecutive patients with foot and ankle problems and their caregivers each completed the OAFQ. The children also completed a Korean version of the KIDSCREEN-52 health related quality of life questionnaire (KIDSCREEN-52 HRQOL). To validate the Korean version of the OAFQ, reliability (child–parent agreement and internal consistency), feasibility (floor and ceiling effects), and construct validity were evaluated, and factor analysis was performed.

**Results:**

In terms of reliability, Cronbach’s α values were > 0.7 in all subscales of the OAFQ (0.765 to 0.901). Child–parent agreement was confirmed by high intraclass correlation coefficients for all subscales (0.791 to 0.863). In terms of construct validity, there were moderate correlations between the subscales of the OAFQ and the subscales of the KIDSCREEN-52 HRQOL. Factor analysis revealed a three-component solution for both the child/adolescent and parent-proxy version, by combining the school and play, and footwear items into one subscale. In terms of feasibility, no floor effects were found for all subscales. However, ceiling effects were observed for the school and play, and emotional subscales for child/adolescent and parent-proxy versions.

**Conclusions:**

The OAFQ was successfully translated and transculturally adapted into the Korean language; the Korean version of the OAFQ represents a reliable and valid instrument for evaluating children’s foot or ankle problems. However, factor analysis suggested the use of a three-subscale questionnaire.

## Background

Infants and adolescents may experience foot and ankle problems for various reasons, such as congenital deformity, secondary deformities due to cerebral palsy, arthritis, or acute injuries. Medical services aim to prevent or correct such deformities and improve physical function using surgery, orthoses, and medication. Assessment of treatment outcomes are usually performed clinically; however, professional assessments performed by health care providers may not accurately reflect the performance of actions commonly required in patients’ everyday lives [1].

Numerous questionnaires have been developed by which to evaluate subjective symptoms, social functioning, quality of life, and improvement after treatment among adults [2]. However, it is difficult to apply an assessment developed for adults to children due to differences between the daily activities of children and adults. Therefore, the Oxford Ankle Foot Questionnaire (OAFQ) for children was developed in 2008 to evaluate foot and ankle issues among pediatric patients [3, 4].

The original English version of the OAFQ has been translated and validated in Dutch, Danish, and Italian [5–7]. Appropriate translations and transcultural adaptations of psychometric instruments according to international guidelines are important to maintain semantic, idiomatic, experiential, and conceptual equivalence to the original version. This could permit the questionnaire’s international use for clinical and research purposes.

Therefore, this study was performed (1) to translate and transculturally adapt the original English version of the OAFQ into a Korean version, and (2) to test the psychometric properties of the Korean OAFQ in terms of reliability (child–parent agreement and internal consistency), feasibility (floor and ceiling effects), construct validity, and factor analysis.

## Methods

### Ethical statements

This study was approved by the institutional review board of our hospital. Informed consent was obtained from all patients’ parents or guardians. Permission for translating the questionnaire was obtained from the copyright holder (Isis Outcomes, Oxford, UK).

### Translation and transcultural adaptation

Translation and transcultural adaptation of the original English version of the OAFQ into a Korean version was conducted according to the established guidelines [8, 9].

1. Forward translation

Two translators (native Korean speakers who are fluent in English) independently translated the English version of the OAFQ into Korean. One translator was an orthopedic surgeon, and the other was a non-medical translator.

2. Reconciliation

A consensus meeting was held with the two translators, three orthopedic surgeons, a registered nurse, and two research assistants. The two preliminary Korean versions of the OAFQ were reconciled into a single Korean version of the OAFQ.

3. Back translation

The reconciled Korean version of the OAFQ was independently back-translated into English by two bilingual Korean-American native English speakers, one of whom was a medical professional translator and the other a non-medical translator. The two back translators had no background information regarding the OAFQ.

4. Harmonization

The back-translated and original versions of the OAFQ were reviewed by a consensus committee composed of three orthopedic surgeons, one registered nurse, two research assistants who specialized in orthopedic scoring systems, a medical professional (a Korean-American dentist), and a non-medical professional (a Korean-American specialist in educational psychology).

Each committee member compared the back-translated and original versions of the OAFQ on an item-by-item basis by scoring the equivalence between the two versions in terms of semantic, idiomatic, experiential, and conceptual equivalence. The equivalence was scored as follows: reject, accept with modification, and accept. After the evaluation of each individual item, all items evaluated as reject or accept with modification were discussed and revised to produce the final Korean version of the OAFQ.

5. Cognitive debriefing

The final version of the Korean OAFQ was preliminarily tested on 15 patients (mean age of 11.9 years; 11 boys and 4 girls) in the outpatient clinic as a previous study recommended 10–40 individuals for pilot testing [8]. A research assistant interviewed the patients and their parents regarding whether they understood each item, and whether they had any difficulties answering the Korean OAFQ. All patients and parents fully understood the items and had no difficulties completing the questionnaire.

6. Proofreading

Any proofreading errors were corrected before the final Korean version of the OAFQ was produced.

### Testing psychometric properties of the Korean OAFQ

Between March and December 2018, 169 consecutive patients aged between 5 and 16 years who visited our outpatient clinics with ankle and foot discomfort and whose parents or caregivers agreed to participate were enrolled in this study. Informed consent was provided by parents or caregivers to a pediatric orthopedic surgeon (KHS). Patients and their caregivers were then asked to separately complete a child/adolescent and a parent-proxy version of the Korean OAFQ and the Korean version of the KIDSCREEN-52 health related quality of life (KIDSCREEN-52 HRQOL) questionnaire for children and adolescents under the supervision of a research assistant.

The OAFQ includes 15 items, of which 14 are grouped into three subscales: physical (6 items), emotional (4 items), and school and play (4 items). The final item queries patients’ satisfaction with the footwear they are able to wear. The KIDSCREEN-52 HRQOL questionnaire consists of 52 items that assess the following 10 HRQOL dimensions: physical wellbeing (5 items), psychological wellbeing (6 items), moods and emotions (7 items), social support and peers (6 items), parent relations and home life (6 items), self-perception (5 items), autonomy (5 items), school environment (6 items), social acceptance (3 items), and financial resources (3 items) [10]. Each item in the OAFQ and KIDSCREEN-52 HRQOL is rated using a 5-point Likert-type response scale ranging from 0 to 4. A high score represents better function.

According to COnsensus-based Standards for the selection of health status Measurement INstruments (COSMIN) guidelines, we validated the Korean version of the OAFQ in terms of reliability (child–parent agreement and internal consistency), feasibility (floor and ceiling effects), and construct validity, and factor analysis was performed on data collected using the instrument [11, 12].

We hypothesized that there would exist significant correlations between the physical subscale of the OAFQ and the physical wellbeing subscale of the KIDSCREEN-52 HRQOL, between the school and play subscale of the OAFQ and the social support and peers subscale of the KIDSCREEN-52 HRQOL, and between the emotional subscale of the OAFQ and the psychological wellbeing subscale of the KIDSCREEN-52 HRQOL.

### Statistical analysis

Cronbach’s α was used to assess internal consistency; a value ≥0.7 was considered satisfactory [13]. Child–parent consistency for each domain’s score was assessed using the intraclass correlation coefficient (ICC) [14]. Construct validity between the OAFQ subscale and the KIDSCREEN-52 HRQOL subscale was evaluated using Pearson’s correlation coefficient. Pearson’s correlation coefficients were characterized as weak (r<0.3), moderate (0.3≤r<0.7), or strong (r≥0.7). Principal components analysis with orthogonal (varimax) rotation was performed using the raw scores [15]. Only factor loadings > 0.50 were considered indicative of item loading. The suitability of the factor analysis was determined by the Kaiser-Meyer-Olkin (KMO) measure of sampling adequacy and Bartlett’s sphericity test. A KMO value of > 0.6 was considered acceptable for the factor analysis [16]. Floor and ceiling effects were considered present if > 15% of the respondents generated the highest or lowest possible scores [17].

Statistical analyses were performed using SAS 9.4 (SAS Institute, Cary, NC, USA) and SPSS software for Windows (version 25.0; IBM Corp., Armonk, NY, USA). All statistics were two-tailed, with a *p*-value < 0.05 considered statistically significant.

## Results

### Translation and transcultural adaptation

During the harmonization process, one item (question 3: “Has it been difficult to stand up for long periods?”) was scored as accept with modification. The two back translations of the sentence were “Have you ever felt that standing for a long time is not easy?” and “Have you ever felt tired from standing a long time?” After discussion, the phrase was revised to “Have you ever felt tired from standing a long time?” in the final Korean version of the OAFQ.

### Psychometric properties of the Korean OAFQ

Among the 169 patients (10.8 ± 3.3 years; 102 boys and 67 girls) enrolled in this study, all 169 children and 164 parents or caregivers completed the questionnaire. It required approximately 20 min to complete both OAFQ and KIDSCREEN-52 HRQOL questionnaires. No missing items occurred. The patients’ diagnoses included flat feet, trauma, deformity of ankle and foot, osteochondral lesion of the talus, Sever’s disease, Achilles tendon problems, tumor, hallux valgus, accessory navicular syndrome and others (Table 1). The average and range, by subscale, of the questionnaires as completed by children and parents are presented in Table 1.

**Table 1:** Summary of data

Total no. of patients			169	
Mean age (years)			10.8 (SD, 3.3)	
Gender (M/F)			102 / 67	
Diagnosis (no. of patients)				
Flat feet			50	
Trauma			39	
Osteochondral lesion of talus			26	
Deformity of ankle or foot			9	
Sever’s disease			9	
Achilles tendon problem			8	
Tumor			7	
Hallux valgus			6	
Accessory navicula syndrome			6	
Other (osteomyelitis, Kohler disease, and juvenile idiopathic arthritis)			9	
	Child		Parent	
	Mean (SD)	Range	Mean (SD)	Range
Physical	2.6 (0.9)	0.2 to 4	2.5 (0.9)	0.2 to 4
School & play	3.0 (1.0)	0 to 4	3.0 (1.0)	0 to 4
Emotional	3.5 (0.6)	0.6 to 4	3.6 (0.6)	0.6 to 4
Foot wear	3.1 (1.2)	0 to 4	3.1 (1.1)	0 to 4
SD, Standard deviation				

In terms of reliability, Cronbach’s α values of the child and parent-proxy versions of the physical, school and play, and emotional subscales were > 0.7 (0.765 to 0.901). All subscales showed high ICCs between the child and parent questionnaires (Table 2).

**Table 2:** Reliability coefficients and agreement for internal consistency and child- and parent-reported scores

	Internal consistency (Cronbach’s α)		Child-parent (ICC)	95% CI
	Child	Parent		
Physical	0.867	0.901	0.801	0.738 to 0.850
School & play	0.869	0.858	0.863	0.817 to 0.897
Emotional	0.765	0.804	0.822	0.764 to 0.866
Foot wear	–	–	0.791	0.726 to 0.843
Total	0.884	0.905	0.868	0.825 to 0.902

In terms of construct validity, there were moderate correlations between the school and play subscale of the OAFQ and the social support and peers subscale of the KIDSCREEN-52 HRQOL, and between the emotional subscale of the OAFQ and the psychological wellbeing subscale of the KIDSCREEN-52 HRQOL. There was weak correlation between the physical subscale of the OAFQ and the physical wellbeing subscale of the KIDSCREEN-52 HRQOL for the parent version, but there was no significant correlation for the child/adolescent version (Table 3).

**Table 3:** Correlation between the OAFQ and KIDSCREEN-52 HRQOL subscales

KIDSCREEN	OAFQ-Child (*r*)			OAFQ-Parent (*r*)		
	Physical	School and play	Emotional	Physical	School and play	Emotional
Physical wellbeing	0.127	**0.283**	**0.275**	**0.219**	**0.246**	0.131
Psychological wellbeing	**0.374**	**0.318**	**0.435**	**0.228**	**0.255**	**0.278**
Moods and emotions	**0.216**	**0.372**	**0.233**	**0.211**	**0.362**	**0.305**
Social support and peers	**0.315**	**0.393**	**0.396**	0.192	**0.317**	**0.438**
Parent relation and home life	**0.426**	**0.326**	**0.344**	**0.298**	**0.243**	**0.206**
Self-perception	**0.284**	**0.366**	**0.300**	0.231	**0.277**	**0.305**
Autonomy	**0.469**	**0.418**	**0.383**	**0.354**	**0.318**	**0.364**
School environment	**0.376**	**0.376**	**0.366**	**0.248**	**0.345**	**0.429**
Social acceptance (bullying)	0.136	0.154	0.057	0.183	0.197	0.284
Financial resources	0.244	0.135	0.217	0.171	0.138	0.188

Bold indicates *p-*value < 0.05

Factor analysis suggested three-subscale questionnaire, as opposed to the original four subscales of the OAFQ. However, most items showed similar loadings according to their original subscales for both the child and parent questionnaires, except for the school and play, and footwear items, as follows: component 1 corresponded to all physical items, component 2 corresponded to all emotional items, and component 3 corresponded to all school and play, and footwear items (Table 4).

**Table 4:** Factor analysis of Oxford ankle foot questionnaire

Items	Factor loading					
	OAFQ-Child			OAFQ-Parent		
	Component 1	Component 2	Component 3	Component 1	Component 2	Component 3
Physical 1	**0.81**	0.26	0.14	**0.84**	0.10	0.24
Physical 2	**0.61**	0.56	0.11	**0.72**	0.08	0.48
Physical 3	**0.70**	0.12	0.30	**0.77**	0.20	0.30
Physical 4	**0.74**	0.18	0.13	**0.77**	0.16	0.19
Physical 5	**0.77**	0.24	0.09	**0.73**	0.17	0.25
Physical 6	**0.66**	0.05	0.34	**0.69**	0.37	0.08
Emotional 1	0.16	**0.90**	0.08	0.31	0.12	**0.86**
Emotional 2	0.18	**0.90**	0.08	0.31	0.11	**0.87**
Emotional 3	0.16	**0.83**	0.17	0.23	0.24	**0.78**
Emotional 4	0.28	**0.60**	0.16	0.21	0.34	**0.53**
School & play 1	0.15	0.01	**0.83**	0.16	**0.78**	0.11
School & play 2	0.15	0.17	**0.75**	0.30	**0.78**	0.02
School & play 3	0.29	0.23	**0.67**	0.17	**0.74**	0.12
School & play 4	0.09	0.13	**0.62**	0.04	**0.72**	0.22
Foot wear	0.12	0.03	**0.52**	0.09	**0.52**	0.18

Bold numbers represent the most appropriate components responding to each item

In terms of feasibility, no floor effects were found for all subscales. However, ceiling effects were observed for the school and play subscale for 51 children (30.2%) and 52 parents (30.8%), and the emotional subscale for 79 children (46.7%) and 86 parents (50.9%).

## Discussion

In this study, translation and transcultural adaptation of the OAFQ into Korean was undertaken, using an established guideline. No subscales exhibited floor effects; however, the emotional and the school and play subscales in both child/adolescent and parent-proxy versions exhibited ceiling effects. All subscales showed satisfactory internal consistency in terms of Cronbach’s α, and child–parent reliability was excellent. The subscales of the Korean OAFQ showed were moderately correlated with those of the KIDSCREEN-52 HRQOL questionnaire. Factor analysis revealed a three-component solution for both the child and parent questionnaires, which differed from the original four subscales because the school and play subscale and the footwear subscale loaded upon the same component.

During the translation and transcultural adaptation process, question 3 (“Has it been difficult to stand up for long periods?”) was disputed. We believe that the word “difficult” can be interpreted in a variety of ways; therefore, the text was changed to “felt tired” instead of “difficult” after discussion. In addition, question 8 (“Has your foot or ankle stopped you playing in the park or outside?”) was modified to use an appropriate verb tense of a similar meaning.

In this study, all subscales in the child/adolescent and parent-proxy version exhibited no floor effects, similar to the Italian and the Danish versions. However, the school and play, and emotional subscales in both child/adolescent and parent-proxy version exhibited ceiling effects, consistent with the findings of previous studies. The Italian version exhibited a ceiling effect for the school and play subscale in both child/adolescent and parent-proxy versions, and for the emotional subscale in the child/adolescent version [7]. The Danish version demonstrated similar ceiling effects to the Korean version [6]. We suggest two possible reasons for these findings. First, foot and ankle problems might not affect school and play, and emotional aspects to the same extent as physical aspects. Second, the disease severity of the patients may affect the results. In the current study, most patients (92.9%) received conservative treatment; only 12 patients received an operation.

All subscales showed Cronbach’s α values of > 0.7 (0.765 to 0.901) in the Korean version, while the emotional subscale in the Danish version for children showed Cronbach’s α values of 0.67 [6]. The remaining subscales in the Danish version exhibited Cronbach’s α values of > 0.7. Similarly, the physical, and school and play subscales in the Dutch version exhibited satisfactory internal consistency, with Cronbach’s α values of > 0.7, but the emotional subscale did not do so in both children (0.60) and parents (0.67) [5]. In the Italian version, as with the Korean version, the school and play and emotional subscales showed Cronbach’s α values of ≥0.7 [7].

Regarding construct validity, as we hypothesized, there were moderate correlations between the school and play subscale of the OAFQ and the social support and peers subscale of the KIDSCREEN-52 HRQOL, and between the emotional subscale of the OAFQ and the psychological wellbeing subscale of the KIDSCREEN-52 HRQOL. There was weak correlation between the physical subscale of the OAFQ and the physical wellbeing subscale of the KIDSCREEN-52 HRQOL for the parent version, but there was no such correlation for the child/adolescent version, contrary to the Dutch version [5]. The reason for this discrepancy might be that the physical wellbeing subscale of the KIDSCREEN-52 HRQOL contains items that address general aspects of physical function, but the physical subscale of the OAFQ contains items confined to ankle and foot problems. In this study, the correlation between the OAFQ and KIDSCREEN-52 HRQOL was higher for the child/adolescent version than for the parent-proxy version. Parents’ perceptions of their child’s condition do not necessarily reflect their child’s perceptions of his or her own condition. This finding emphasizes that it is important for parents to ask their child about his or her symptoms and HRQOL because disagreements may exist regarding quality of life as assessed by children themselves versus their parents [18].

In factor analysis, component 1 corresponded to the physical item; component 2 the school and play item, and component 3 all emotional items and the footwear item. These results suggest that the number of subscales could be reduced to three, as opposed to the four subscales of the original OAFQ. A Korean OAFQ with three subscales could be developed by combining the school and play, and footwear items into one subscale.

Some limitations of our study need to be addressed. First, test-retest reliability was not assessed in this study. Future research is warranted to investigate this measurement property. Second, the responsiveness of the Korean OAFQ was not evaluated in this study, which is an aspect of validity. Therefore, further study of the responsiveness of the Korean OAFQ is required.

## Conclusion

In conclusion, the OAFQ was translated and transculturally adapted into Korean in accordance with international guidelines. The Korean version of the OAFQ is a reliable and valid instrument for assessing children’s foot or ankle problems. However, the Korean version of the OAFQ could be reformulated by dividing it into three subscales, as suggested by factor analysis.

## Data Availability

The data set supporting the conclusion of this article is available on request to the corresponding author.

## Abbreviations

OAFQ: Oxford Ankle Foot Questionnaire
ICC: Intraclass correlation coefficient
HRQOL: Health related quality of life
